# Continuous Suprainguinal Fascia Iliaca Block for Postoperative Analgesia Management After Hip and Knee Arthroplasty Surgeries: A Report of Two Cases

**DOI:** 10.7759/cureus.77016

**Published:** 2025-01-06

**Authors:** Ufuk Demir, Öztürk Taşkın, Ayşe Yılmaz, Büşra Tanyıldızı Küçük, Zahide Doğanay

**Affiliations:** 1 Anesthesiology and Reanimation, Kastamonu University Faculty of Medicine, Kastamonu, TUR

**Keywords:** block catheter, continous block, continuous suprainguinal fascia iliaca block, facial plan block, hip arthroplasty, knee arthroplasty, postoperative analgesia, regional anesthesia, suprainguinal fascia iliaca block, suprainguinal fascia iliaca block catheter

## Abstract

Knee and hip arthroplasty are common orthopedic procedures that are frequently associated with moderate to severe postoperative pain. Regional anesthesia techniques play a crucial role in multimodal analgesia strategies for managing postoperative pain.

The suprainguinal fascia iliaca (SIFI) block is an effective regional anesthesia technique frequently used for postoperative analgesia in lower extremity surgeries, including knee and hip arthroplasty. These techniques can be safely applied either as a single-shot injection or via continuous catheter insertion.

In this case report, we describe our experience with the use of continuous SIFI block for postoperative analgesia in knee and hip arthroplasty.

## Introduction

Both knee and hip arthroplasty surgeries are widely performed worldwide [1]. The postoperative pain associated with both surgeries is reported to range from moderate to severe [2,3]. Regional anesthetic techniques are widely used as an essential part of multimodal analgesia in postoperative pain management [4].

The fascia iliaca compartment is a potential space located anteriorly between the fascia iliaca and posteriorly between the iliacus and iliopsoas muscles. The fascia iliaca block was first described by Dalens et al., and the suprainguinal fascia iliaca (SIFI) block was later introduced by Stevens et al. [5,6]. The SIFI block is expected to affect the branches of the lumbar plexus, including the femoral, obturator, and lateral cutaneous nerves, as all these nerves pass through the fascia iliaca compartment during part of their anatomical course. Although the lumbar plexus does not provide complete innervation to the knee and hip joints, it plays a critical role in their sensory and motor functions. Indications for the SIFI block include femoral neck fractures, hip and knee surgeries, above-knee amputations, and cast applications for femoral fractures in pediatric patients [7,8,9].

SIFI block has been reported in various studies for knee and hip arthroplasty surgeries [3,7,10]. Additionally, in a cadaveric study and another study involving hip fracture patients, the authors demonstrated the use of catheters for continuous SIFI blocks [11,12]. However, continuous SIFI block has not been applied before in knee or hip arthroplasty.

In this paper, we aim to present our continuous SIFI block applications, performed for the first time in the literature to the best of our knowledge for postoperative analgesia in knee and hip arthroplasty surgeries.

## Case presentation

Case 1

**Figure 1:**
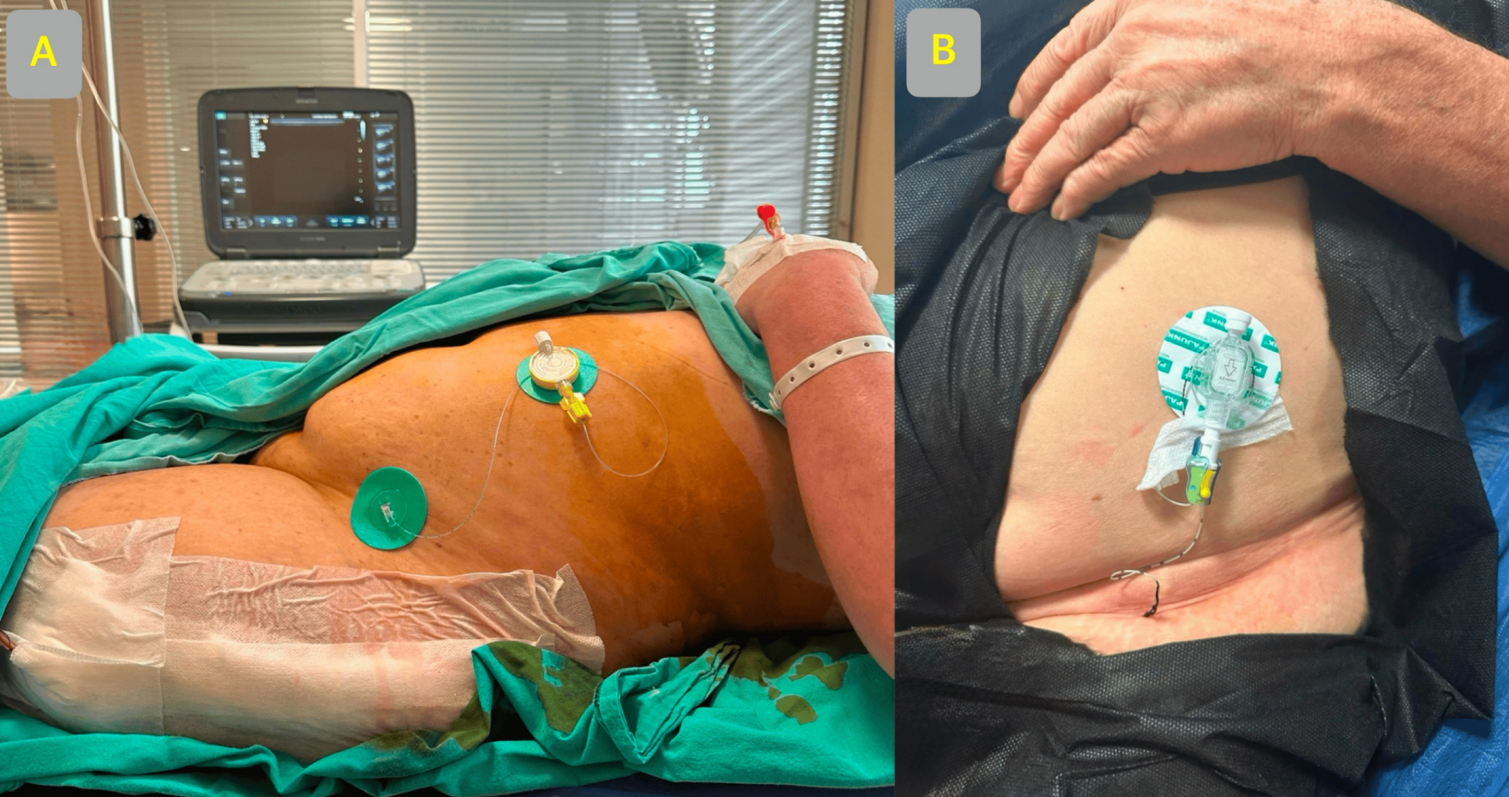
Application of continious suprainguinal fascia iliaca block. (A) hip arthroplasty; (B) knee arthroplasty.

A 67-year-old female patient, 160 cm in height, weighing 88 kg, with a body mass index (BMI) of 34, diagnosed with chronic obstructive pulmonary disease (COPD) and classified as American Society of Anesthesiologists (ASA) physical status 3, underwent left total hip arthroplasty with a lateral incision under spinal anesthesia. The surgery lasted 115 minutes, and no complications were encountered during the surgery. Postoperatively, the SIFI block was performed with ultrasound guidance using 60 mL of a local anesthetic mixture (0.5% bupivacaine 20 mL and 40 mL saline) in the post-anesthesia care unit (PACU). Subsequently, a catheter (Epifix® 18 G Tuohy needle with 20 G epidural catheter, Egemen, Turkey) was placed, as shown in Figure 1A.

**Figure 2:**
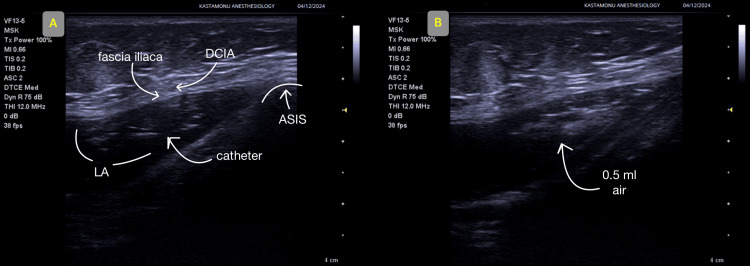
Ultrasound images. (A) continuous suprainguinal fascia iliaca block placement; (B) confirmation of catheter position. The same method was applied in both cases. DCIA: deep circumflex iliac artery, LA: local anesthetic solution, ASIS: anterior superior iliac spine.

The catheter placement was confirmed using 0.5 mL of air injection, as shown in Figure 2A and Figure 2B. 

The same dose and volume of the local anesthetic mixture were administered every 24 hours via the block catheter, along with intravenous paracetamol 1 gm every six hours, for postoperative analgesia. The patient's 1st, 6th, 12th, and 24th-hour Visual Analog Scale (VAS) scores during movement and rest, presence of motor block, walking times, and rescue analgesic needs were monitored and recorded for five days.

In the postoperative period, rescue analgesia was planned when the VAS score exceeded 4, and 25 mg of meperidine was planned as a rescue analgesic. During the follow-up period, the VAS score did not exceed 4, the motor block was not observed, and rescue analgesics were not required. The patient was discharged on the fifth day, with the catheter removed without complications. Written informed consent was obtained from the patient for all procedures.

Case 2

A 56-year-old female patient, 157 cm in height, weighing 80 kg, with a BMI of 32, diagnosed with controlled hypertension and classified as ASA physical status 2, underwent left total knee arthroplasty with an anterior incision under spinal anesthesia. The surgery lasted 135 minutes, and no intraoperative complications were encountered. The surgery lasted 135 minutes, and no complications were encountered during the surgery. Postoperatively, the SIFI block was performed with ultrasound guidance using 60 mL of a local anesthetic mixture (0.5% bupivacaine 20 mL and 40 mL saline) in the PACU. Subsequently, a catheter (Contiplex® D Set NRFit®, Braun, Germany) was placed (Figure 1B). The catheter placement was confirmed using 0.5 mL of air injection, as shown in Figure 2A and Figure 2B.

For postoperative analgesia, the same dose and volume of the local anesthetic mixture were administered every 24 hours via the block catheter, along with intravenous paracetamol 1 gm every six hours. VAS scores during movement and rest, presence of motor block, and rescue analgesic needs were monitored and recorded for 5 days.

In the postoperative period, rescue analgesia was planned when the VAS score exceeded 4, and 25 mg of meperidine was planned as a rescue analgesic. During the follow-up period, the VAS score did not exceed 4, the motor block was not observed, and rescue analgesics were not required. The patient was discharged on the fifth day, with the catheter removed without complications. Written informed consent was obtained from the patient for all procedures.

Continuous Suprainguinal Fascia Iliaca Block Technique

For the SIFI block, patients were placed in the supine position, and all procedures were performed under aseptic conditions. A 13.5 MHz linear probe of an ultrasound device (Siemens, Mountain View, CA, USA) was used for the SIFI block and catheter placement. For the SIFI block, following negative aspiration, 60 mL of local anesthetic was injected into the compartment between the iliac fascia and iliacus muscle in the suprainguinal region using an in-plane technique. The spread of the local anesthetic was confirmed via ultrasound. After the block was administered, the catheter was advanced through the needle, and its position was verified by injecting 0.5 mL of air.

## Discussion

Postoperative analgesia is an important part of anesthesia management that should be planned by anesthesiologists. In recent years, it has been recommended to reduce opioid consumption and use multimodal analgesia methods in postoperative analgesia management. Regional anesthetic techniques play a major role in multimodal analgesia planning [1,3,4].

Regional anesthetic techniques include central blocks (epidural anesthesia, spinal anesthesia, and combined spinal epidural anesthesia), peripheral nerve blocks, and fascial plane blocks. Although central blocks are considered the most effective methods in postoperative analgesia, they have recently been less frequently used due to the many major complications [1,4]. One of the reasons for this is that the use of ultrasound in regional anesthesia has increased, and peripheral nerve block and fascial block techniques have developed greatly in recent years [1,3].

Many regional anesthesia techniques have been applied for postoperative analgesia in lower extremity surgeries. These include peripheral nerve and plexus blocks, such as blocking the lumbar plexus, femoral nerve, obturator nerve, and sciatic nerve at different anatomical levels. These techniques can be applied separately or together [1,3,4]. In addition, these block methods can be applied with ultrasound as a single shot or can be safely applied continuously by placing a catheter. In patients with catheter placement, local anesthetic injections can be administered either as continuous infusions or intermittent boluses. Among the advantages of catheter applications in peripheral nerve blocks reducing pain scores for a longer period of time, reducing opioid use and related side effects, reducing sleep disorders, and increasing patient satisfaction have been reported [13]. It has also been reported that inadequate postoperative analgesia increases the incidence of chronic pain [14].

SIFI block is also a regional anesthesia technique applied for postoperative analgesia in lower extremity surgery. It has been reported that SIFI block provides good analgesia for approximately the first 24 hours postoperatively [3]. The most severe postoperative pain is in the first 24 hours of pain, but severe pain continues for up to 72 hours [2]. We aimed to prolong the analgesia duration of the SIFI block by administering intermittent local anesthetic boluses via the catheter.

## Conclusions

In these case reports, we aimed to prolong the duration of analgesia by implementing SIFI block catheters instead of single injections in knee and hip arthroplasty surgeries. We believe that the application of the continious SIFI block is a simple and safe technique. However, we suggest that the efficacy and safety of continuous SIFI block applications in knee and hip arthroplasty surgeries should be investigated through randomized controlled trials.
